# How to talk about death? A cross-sectional survey on patients’, informal caregivers’ and health care professionals’ views in the setting of allogenic hematopoietic stem cell transplantation

**DOI:** 10.1007/s00277-026-06981-7

**Published:** 2026-04-02

**Authors:** Lisanne Preuss, Steffen T. Simon, Marco Herling, Udo Holtick, Alinda Reimer, Berenike Schoerger, Sukhvir Kaur, Jithmi Weliwitage, Martin Hellmich, Michael Hallek, Carolin Schepers, Johann Ahn, Christof Scheid, Roland Schroers, Georg-Nikolaus Franke, Raymond Voltz, Anne Pralong

**Affiliations:** 1 Faculty of Medicine and University Hospital, Department of Palliative Medicine, University of Cologne, Cologne, Germany; 2 Faculty of Medicine and University Hospital, Center for Integrated Oncology Aachen-Bonn-Cologne-Duesseldorf (CIO ABCD), University of Cologne, Cologne, Germany; 3 Faculty of Medicine and University Hospital, Center for Health Services Research (ZVFK), University of Cologne, Cologne, Germany; 4 Faculty of Medicine and University Hospital, Department I of Internal Medicine, University of Cologne, Cologne, Germany; 5 Faculty of Medicine and University Hospital Cologne, Institute of Medical Statistics and Computational Biology (IMSB), University of Cologne, Cologne, Germany; 6 Department of Hematology, Cellular Therapy, Hemostaseology, and Infectious Diseases, University of Leipzig, Leipzig, Germany; 7 Department of Hematology, Oncology and Tumor Immunology, Campus Virchow-Klinikum (CVK), Charité – Universitätsmedizin Berlin, Berlin, Germany; 8 Knappschaft Kliniken- University Hospital Bochum, Department of Hematology and Oncology, Ruhr University Bochum, Bochum, Germany; 9 Comprehensive Cancer Center Central Germany (CCCG) Leipzig-Jena, Jena, Germany

**Keywords:** End-of-life communication, Stem cell transplantation, Life threat, Palliative care, Health care professionals

## Abstract

Allogeneic hematopoietic stem cell transplantation (allo-HSCT) is a potentially curative treatment, which carries a high risk of complications and mortality, highlighting the necessity of discussions about life threat. This study explores the perspectives of patients, informal caregivers (ICs), and healthcare professionals (HCPs) regarding the timing, content, and challenges of these discussions. Multicenter cross-sectional survey in comprehensive care centers across Germany, involving patients with allo-HSCT, ICs, and HCPs. Questionnaires assessed perceived concerns about life threat, communication needs, and attitudes toward death. Statistical analyses included descriptive statistics, group comparisons, and multivariate logistic regressions. A total of 61 patients, 31 ICs and 125 HCPs participated in this study. Patients and ICs had a high need for discussion about life threat at diagnosis, which was corroborated by HCPs. At further time points in the transplant trajectory, there were more discrepancies between patients‘/ICs‘ and HCPs’ perceptions towards conversations about life threat. While 61.7% of patients preferred having their ICs present during discussions, ICs often felt overlooked, with only 50% finding conversations with HCPs helpful. HCPs’ avoidance of death was associated with a reduced likelihood of reporting a need for conversation in patients in the event of severe complications. These findings highlight a discrepancy between patients’ and ICs’ preferred timing for conversations about life threat and HCPs’ perceptions of when patients actually express such a need. To bridge this gap, earlier and ongoing conversations are essential. Training programs should address HCPs’ discomfort in discussing prognosis and improve interdisciplinary teamwork to standardize end-of-life communication.

**Trial registration number**: DRKS00027290 (German Clinical Trials Register) on 10.01.2022.

**Supplementary Information:**

The online version contains supplementary material available at 10.1007/s00277-026-06981-7.

## Introduction

Allogenic hematopoietic stem cell transplantation (allo-HSCT) is a potentially curative treatment option for malignant and non-malignant diseases, it carries significant risks, including life-threatening complications such as Graft-versus-Host-Disease (GvHD) [1] and a heightened probability of infections [2–4]. The mentioned complications as well as a relapse of the underlying disease increase the risk of a rapid transition from a potentially curative treatment to an end-of-life (EOL) situation for patients, with a five-year survival, all diagnoses included, at 50% [5]. Allo-HSCT recipients therefore are dealing with the simultaneity of being cured as well as a relevant risk of dying [6].

Consequently, communication about life threat and EOL is a critical component of the treatment process, yet studies show that these discussions are often delayed, lack clarity, or do not adequately involve patients in decision-making [7, 8]. The current scientific discourse emphasizes the need to place greater focus on communication, as existing communication aids and training programs from oncology are not tailored to the specific requirements and unique challenges of allo-HSCT [9–11].

Wright et al. examined the mental health of patients and their families in relation to EOL conversations, as well as the negative impact aggressive medical interventions can have [12]. Their findings revealed that only 37% of patients had EOL discussions with their healthcare professionals (HCPs), while in the study by Odejide et al., 55.9% of HCPs reported having these discussions “too late”. Delayed EOL conversations can result in the continuation of aggressive treatments, such as chemotherapy, which may no longer provide clinical benefit and could potentially be avoided through earlier communication, as well as utilization of palliative care (PC) [13].

Despite the importance of these conversations, there is no standard practice regarding when or how these sensitive topics should be addressed in the field of allo-HSCT and little is known about how patients, informal caregivers (IC) and HCPs perceive the timing, content, and adequacy of such discussions, and whether their perspectives align [9–11, 14–16].

To address this knowledge gap, we conducted a multicenter cross-sectional survey including these key stakeholders. As life threat communication in allo-HSCT is inherently shared, comparing these perspectives helps identify common needs and mismatch relevant for patient-centered care.

## Methods

This survey was developed within the research project “Allo-PaS” (Palliative-supportive management in allo-HSCT; registration number: DRKS00027290, German Clinical Trials Register) [17]. The findings of this survey will serve as an empirical basis for the subsequent development of a structured palliative-supportive care intervention.

### Study design

Patients and ICs were recruited through a multicentered network of comprehensive cancer centers (CCCs) across Germany. Of ten invited centers, seven recruited patients and/or ICs (Online Resource 12). Recruitment procedures were defined centrally within the Allo-PaS project: patients and ICs were informed about the study via standardized study flyers and direct invitation by treating HCPs in the respective HSCT departments. While the recruitment strategy was consistent across centers implementation depended on local organizational structures and feasibility.

After confirming their participations, the individuals were contacted by the Allo-PaS research team for an initial screening by telephone to check the inclusion criteria (Table 1).

After consenting, paper-pencil questionnaires were sent out with the possibility of telephone support.

HCPs were recruited through an online survey that was sent to five German tertiary hospitals as part of.

CCCs (Online Resource 12). All five centers recruited HCPs. A designated employee from each HSCT department distributed the survey to HCPs within their respective centers. Data collection took place between January and February 2023 using LimeSurvey^®^.

**Table 1:** Inclusion and exclusion criteria for survey participants

Participants	Inclusion criteria	Exclusion criteria
Patients	● Deemed eligible for allo-HSCT or having undergone allo-HSCT ● ≥ 18 years ● Informed consent	● Insufficient German language skills ● Severe physical impairment ● Severe cognitive impairment ● Undergone allo-HSCT more than 1 year ago ● Not deemed eligible for allo-HSCT or have not undergone allo-HSCT
Informal caregivers	● Informal caregivers of a patient with allo-HSCT ● ≥ 18 years ● Informed consent	● Non-sufficient German language skills ● Severe physical impairment ● Severe cognitive impairment
HCP	● Belonging to a relevant professional group (physicians, nurses, psycho-oncologists) ● ≥ 18 years ● Informed consent	● Non-sufficient experience in caring for patients with allo-SCT ● Non-sufficient German language skills

### Development

The survey consists mainly of self-developed questions (Online Resource 1) as well as of standardized questionnaires. Open-ended questions allow for free-text responses (Online Resource 2).

The self-developed questions for patients and ICs assess concerns and conversation needs surrounding the life threat experienced by patients, focusing on timing, setting and preferred discussion partners.

The HCPs’ questionnaire addresses the perceived emotional and conversational needs of their patients and ICs, as well as compares HCPs‘ preferred with their actual practices when discussing life threat with patients and ICs.

The questionnaires were developed using a multistep process. An initial item pool was based on Allo-PaS research findings [17], a literature review [18] and expert discussions. The draft was reviewed by an interdisciplinary expert group to assess content validity, clarity, and redundancy. The revised version was pilot tested with researchers, HCPs, patients, ICs to evaluate comprehensibility and response burden, leading to linguistic refinements and the exclusion of one questionnaire, due to concerns about excessive burden, raised by patients and ICs.

The standardized questionnaire used to assess Death acceptance in all three participant groups is the subscale *Death Acceptance (DA)* of the German validated *Life Attitude Profile-Revised (LAP-R)* Scale [19, 20]. The *Death Attitude Profile-Revised* (DAP-R) was additionally used in the study population of HCPs [21, 22] and measures attitudes towards death in 32 items, covering 5 dimensions: fear of death, death avoidance, neutral acceptance, approach acceptance and escape acceptance [21, 23] (Online Resource 1).

### Data analysis

Participants’ clinical and sociodemographic characteristics were analyzed using descriptive statistics (frequencies, means, medians). Group comparisons were conducted using T-tests, Mann-Whitney U, Wilcoxon signed-rank, and Kruskal-Wallis tests. Chi-square and Fisher’s exact tests assessed categorical associations, while McNemar’s test compared paired nominal data. For correlations, Spearman’s was used for ordinal/non-normally distributed scores, Pearson’s for normally distributed scores, and Point-Biserial Pearson for binary-continuous relationships. Cramer’s V and Phi (φ) measured categorical associations. Given the relatively small sample size and the number of comparisons, subgroup analyses should be interpreted as exploratory.

To further assess the influence of demographic, professional, and psychometric variables on HCPs’ communication preferences, multivariate logistic regression analyses were conducted using the Backwards Wald method, with independent variables selected a priori based on theoretical and clinical relevance. Multicollinearity was assessed using the variance inflation factor (VIF > 10 indicating substantial multicollinearity).

Effect sizes were calculated and interpreted according to established conventions (Cohen’s r and Cramér’s V: ≥0.10 small, ≥ 0.30 moderate, ≥ 0.50 large; η²: ≥0.01 small, ≥ 0.06 moderate, ≥ 0.14 large). A two-sided p-value < 0.05 was considered statistically significant. Analyses were performed using SPSS (Version 29.0.2.0). Open-ended responses were analyzed using an inductive approach to summarize the data.

## Results

Sociodemographic and clinical data of the participants are presented in Table 2.

A total of 61 patients and 31 ICs from seven centers completed the survey. In total, 138 individuals were referred to the study team for eligibility screening. Due to data protection regulations the total number of patients and ICs initially approached at the participating centers is unknown.

In terms of treatment status, the majority of participants (73.8%, *n* = 45) were in outpatient follow-up care.

A total of 184 HCPs opened the invitation link. Of these, 53 were excluded due to non-response, and 6 did not meet the inclusion criteria. This resulted in a final sample size of 125 HCPs. Eighty-one HCPs filed in the optional DAP-R questionnaire. Due to confidentiality policies, the total number of HCPs who received the survey remains undisclosed, making it impossible to determine the exact response rate.

Unless otherwise indicated, analyses are based on the total sample of patients, ICs, and HCPs.

## Patients

### Concerns about life threat

**Fig. 1:**
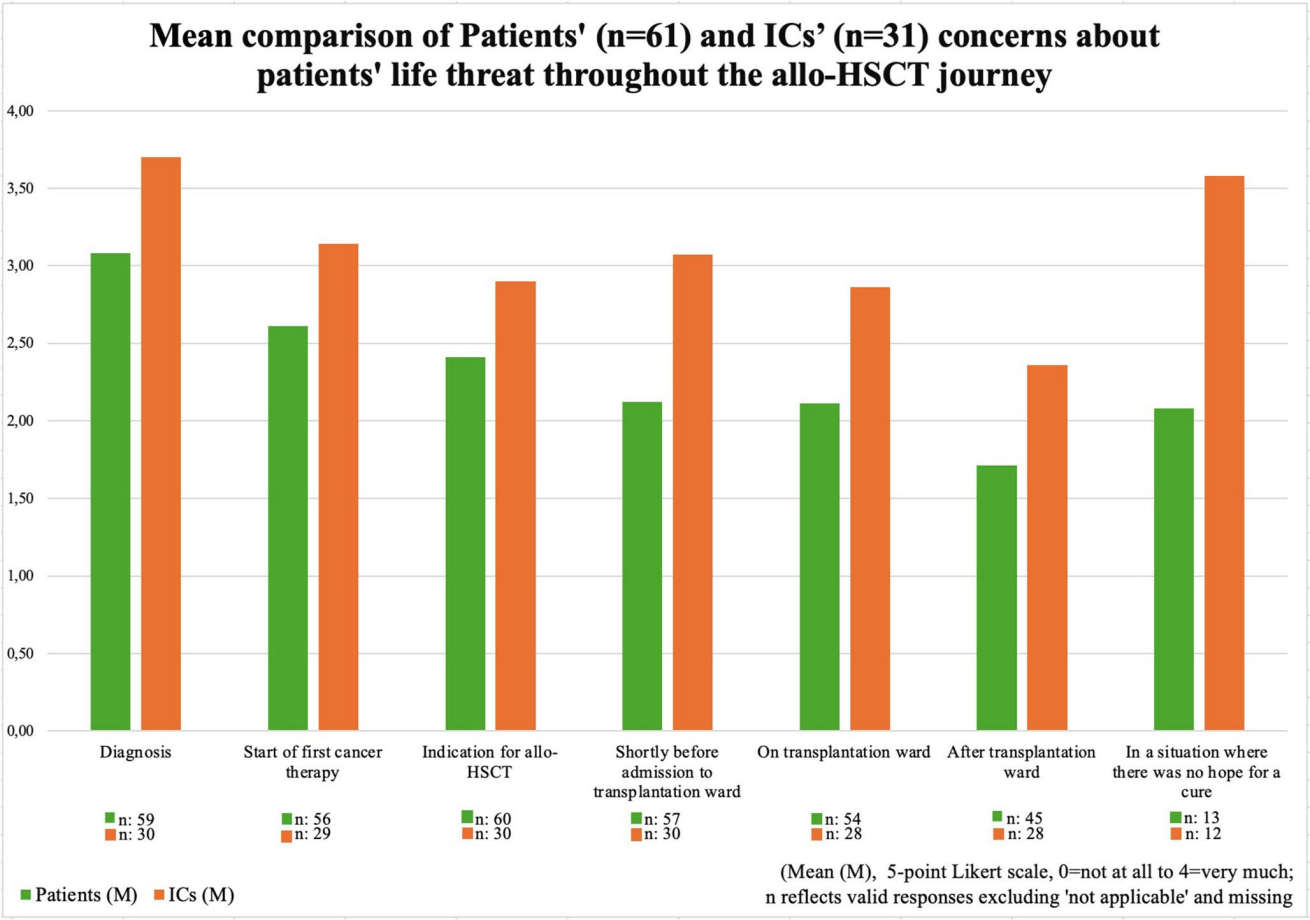
Comparison of Patients’ and ICs’ reported concerns about patients’ life threat throughout the allo-HSCT journey

Patients reported the highest concerns regarding life threat at cancer diagnosis (M = 3.08, SD = 1.28, Max = 4). Further critical moments of concerns included the initiation of hematological therapy and the recommendation for an allo-HSCT (Fig. 1). At post-transplantation, 26.7% (*n* = 12) experienced no concerns at all regarding life threat.

Patients who self-reported having dependent children reported higher concerns after transplantation compared to those without (*p* = 0.038, u = 0.32, Online Resource 7), although this finding should be interpreted with caution given the small subgroup size.

### Discussions on life threat

More than half of the patients (53.3%) preferred an IC present during discussions. The majority of patients (77.0%) preferred hematologists, and 36.1% psychologists, as conversation partners (Online Resource 3). Age and marital status influenced partner preferences, with younger patients favoring psychologists (*r*=-0.321, *p* = 0.012) and nurses (*r*=-0.290, *p* = 0.023), and married/partnered individuals preferring hematologists (*p* = 0.023, Cramér’s V = 0.369), while unmarried, widowed or divorced individuals favored spiritual counselors (*p* = 0.004, Cramér’s V = 0.444, Online Resource 7).

**Fig. 2:**
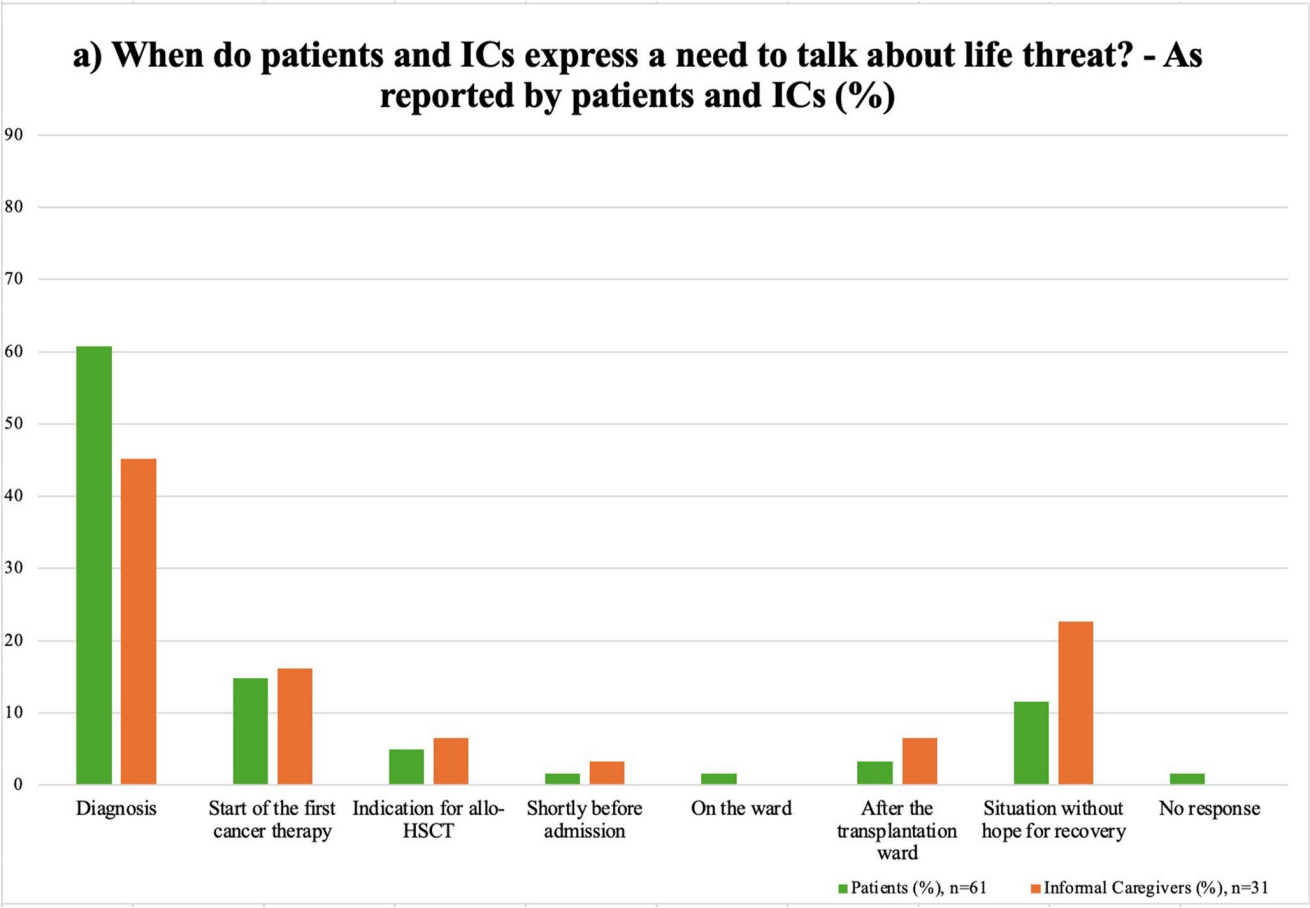

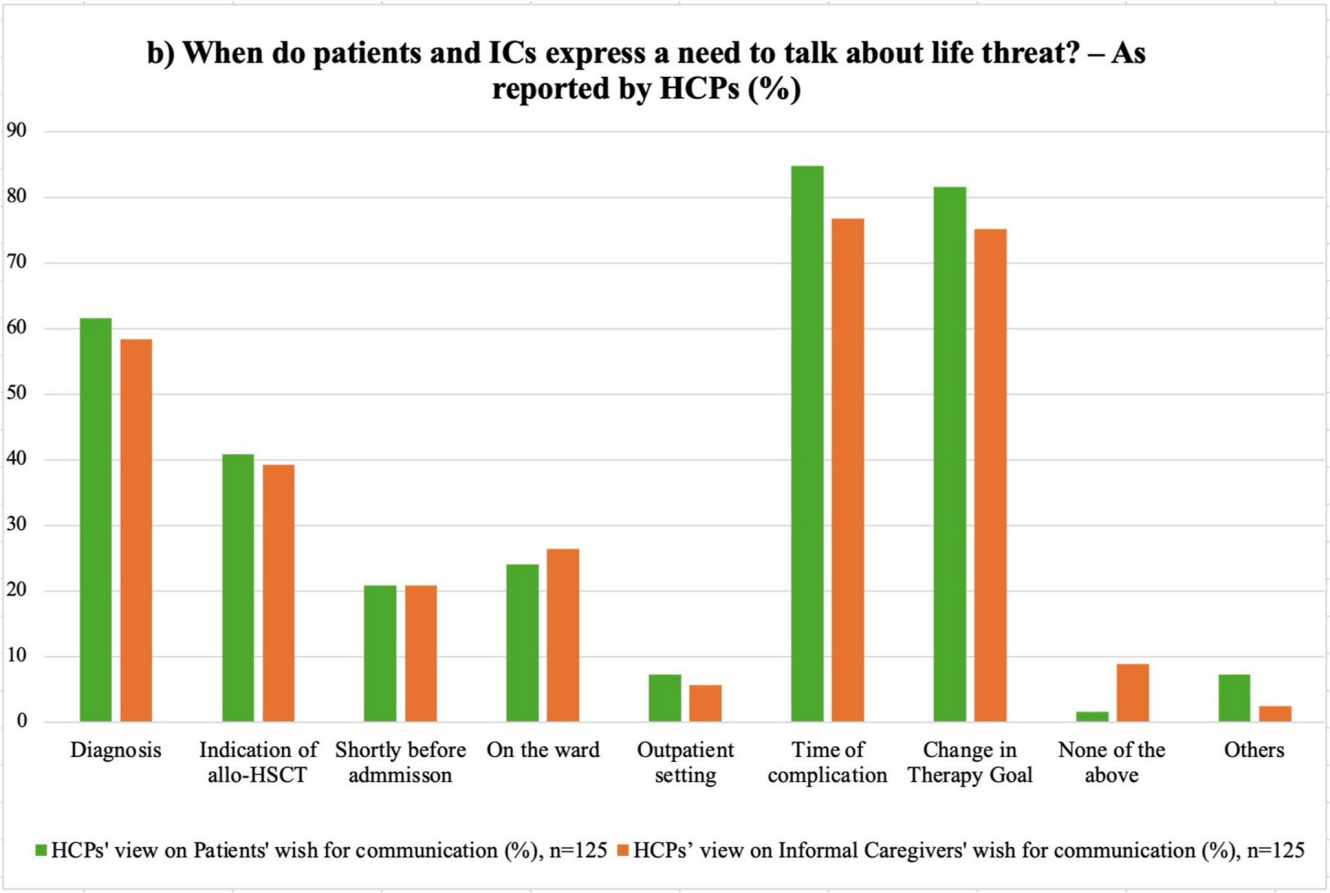
Comparison of patients’ and ICs’ preferences for discussions on life threat at different time points: **a**) Self-reported preferences by patients and ICs vs. **b**) HCPs’ perspective

Diagnosis (60.7%) was the most preferred time for discussion, aligning with the phase of highest patient concerns (Fig. 2a)). Preferences showed a significant association with marital status (*p* = 0.015, Cramér’s V = 0.448), with married individuals favoring discussion at diagnosis and with religiosity, with non-religious patients more frequently preferring later time points, such as indication for allo-HSCT or a hypothetical palliative situation (*p* = 0.032, Cramér’s V = 0.434, Online Resource 7). No significant difference was found between patients and ICs regarding preferred discussion timing (*p* = 0.695, Online Resource 10).

Prognosis was rated the most important discussion topic (M = 3.49, SD = 0.92), though coping (M = 3.03, SD = 1.17), support (M = 2.97, SD = 1.22) and information needs (M = 3.10, SD = 1.15) also held high importance. Female patients rated information needs as significantly more important than male patients (*p* = 0.025, u = 0.286, Online Resource 7).

A conversation about life threat took place for 78.7% (*n* = 48) of patients, with 60.4% (*n* = 29) finding it helpful, while 91.5% (*n* = 54) of patients did not want to seek further support beyond verbal discussions.

**Table 2:** **a**: Demographic and clinical data of patients (*N* = 61), demographic data of ICs (*N* = 31)

	Patients (*N* = 61)	*n* (%) or mean (SD)		Informal caregivers (*N* = 31)	*n* (%) or mean (SD)
**Gender**			**Gender**		
female		20 (32.8)	female		21 (67.7)
Male		41 (67.2)	Male		10 (32.3)
**Age (in years)**		55.1 (± 13.5)	**Age (in years)**		55 (± 12.5)
**Religiosity**			**Religiosity**		
Religious		25 (41)	Religious		14 (45.2)
Non-religious		35 (57.4)	Non-religious		17 (54.8)
Not reported		1 (1.6)	Not reported		0
**Mother tongue**			**Mother tongue**		
German		49 (80.3)	German		28 (90.3)
English		1 (1.6)	English		0
Russian		3 (5.0)	Russian		1 (3.2)
Turkish		2 (3.3)	Turkish		1 (3.2)
Albanian		1 (1.6)	Italian		1 (3.2)
Dutch		1 (1.6)			
Polish		3 (5.0)			
Portuguese		1 (1.6)			
**Family status**			**Family status**		
Single		11 (18)	Single		2 (6.5)
Widowed		3 (4.9)	Widowed		1 (3.2)
Married/Registered Partnership		40 (65.7)	Married/Registered Partnership		25 (80.6)
Divorced/separated		6 (9.8)	Divorced/separated		3 (9.7)
Not reported		1 (1.6)	Not reported		0
**Living situation**			**Living situation**		
Alone		8 (13.1)	Alone		3 (9.7)
With (spouse) partner		39 (63.9)	With (spouse) partner		24 (77.4)
With children		14 (23)	With children		2 (6.5)
With friend		2 (3.3)	With friend		1 (3.2)
With others		7 (11.5)	With others		0
Not reported		0	Not reported		1 (3.2)
**Level of Education**			**Level of Education**		
No qualification		2 (3.3)	No qualification		1 (3.25)
In vocational training		3 (5.0)	In vocational training		1 (3.25)
Vocational training completed		26 (42.6)	Vocational training completed		13 (41.9)
Completion of professional/master craftsman/academic training		11 (18)	Completion of professional/master craftsman/academic training		9 (29)
University Degree		18 (29.5)	University degree		7 (22.6)
Other		0	Other		0
Not reported		1 (1.6)	Not reported		0
**Time of transplantation**			**Relationship to patient**		
(A) About to undergo transplantation		2 (3.3)	Partner		20 (64.5)
(B) Inpatient care due to transplantation		11 (18)	Child		4 (12.9)
(C) Outpatient care after transplantation		45 (73.8)	Parent		4 (12.9)
Missing/Unclear		3 (4.9)	Other		3 (9.7)
**Underlying hematological disease**			**Responsibility for children**		
Acute myeloid leukaemia (AML)		26 (42.6)	Yes		16 (51.6)
Myelo dysplastic syndrom (MDS)		12 (19.7)	No		14 (45.2)
Acute lymphoblastic leukaemia (ALL)		8 (13.1)	Not reported		1 (3.2)
Myeloproliferative neoplasm (MPN)		1 (1.6)	**Number/Age of children**		1.9 (± 0.7) / 19.8 (± 9.5)
Lymphoma		4 (6.6)			
Multiple myeloma		2 (3.3)			
Other		8 (13.1)			
**Responsibility for children**					
Yes		22 (36.1)			
No		37 (60.7)			
Not reported		2 (3.3)			
Number/Age of children		1.7 (±0.6)/ 20.1 (± 11.5)			
**b**: Demographic and professional data of HCPs (*N* = 125)					
			**HCP** ***(N = 125)***	**n (%) or mean (SD)**	
**Gender**					
female				76 (60.8)	
Male				49 (39.2)	
**Age (in years)**				37.47 (± 8.45)	
**Mother tongue**					
German				119 (95.2)	
other				6 (4.8)	
**Professions**					
Physician				58 (46.4)	
Nurse				58 (46.4)	
Psychooncologist				9 (7.2)	
**Health care provision sector**					
HSCT				72 (57.6)	
Oncology				42 (33.6)	
ICU				9 (7.2)	
Not reported				2 (1.6)	
**Experience in the field of allo-HSCT**					
Number of years in the field working with allo-HSCT patients				9.08 (± 7.25)	
Number of allo-HSCT patients treated per year				57.56 (± 54.69)	
Number of patients accompanied during their dying phase per year				10.46 (± 13.32)	

## ICs

### Concerns about life threat

ICs reported the highest concerns about patients’ life threat at cancer diagnosis (M = 3.7, SD = 0.79) and in situations without hope for a cure (M = 3.58, SD = 0.90), while the lowest concerns occurred in outpatient follow-up (M = 2.36, SD = 1.34, Fig. 1). ICs reported significantly higher concerns about life threat than patients at diagnosis (*p* = 0.019), at the start of first cancer therapy (*p* = 0.047), shortly before transplantation (*p* = 0.003), and on the transplantation ward (*p* = 0.021, Online Resource 6). Sociodemographic analyses revealed that female ICs reported higher concerns about life threat on the transplantation ward (*p* = 0.049, u = 0.39), after transplantation (*p* = 0.013, u = 0.44), and in a hypothetical palliative situation (*p* = 0.009, u = 0.95) than male ICs. Younger ICs also reported higher concerns in a hypothetical palliative situation (ρ=−0.594, *p* = 0.042, *n* = 12, Online Resource 8); although results should be interpreted with caution due to the small sample size.

### Discussions on life threat with patients

Conversations were infrequent, with 48.4% (*n* = 15) of ICs rarely and 35.5% (*n* = 11) sometimes addressing the topic with the patient. Only 29.0% (*n* = 9) explicitly discussed EOL. 61.3% (*n* = 19) had not, while 9.7% (*n* = 3) were unsure. Among those who had not discussed EOL, 33.3% (*n* = 6) wanted to have the discussion, 38.9% (*n* = 7) did not, and 27.8% (*n* = 5) were unsure.

Regarding the difficulty of discussing death, only 9.7% (*n* = 3) found it not difficult at all, while 19.4% (*n* = 6) wished for further external support in addressing the topic. 54.8% (*n* = 17) of ICs believed that such discussions did not take away hope, while 32.3% (*n* = 10) did. Older ICs more often felt that discussing life threat would take away hope from their relative than younger ICs (Kruskal-Wallis, *p*=0.038, η²=0.16, Online Resource 8).

Most ICs (83.9%, *n* = 26) had discussed the patient’s will in the event of decisional incapacity.

### Discussions on life threat with HCPs

While 51.6% (*n* = 16) of ICs favored such discussions together with the patient, 32.3% (*n* = 10) could envisage to discuss life threat without the patient. 45.2% (*n* = 14) favored discussing it at diagnosis, and 22.6% (*n* = 7) in a situation without hope for cure. Hematologists (67.6%) were the most preferred conversation partners (Online Resource 3).

Significant associations were found between the favored discussion partner and the ICs’ relationship to the patient, as well as the treatment phase: Spouses preferred to speak with hematologists (*p* = 0.034, Cramér’s V = 0.519), while parents and children showed varied preferences and were more likely to choose nurses (*p* = 0.026, Cramér’s V = 0.595). ICs of patients in outpatient follow-up preferred hematologists (*p* = 0.037, Cramér’s V = 0.466).

The most important discussion topic was prognosis (M = 3.71, SD = 0.64) though coping (M = 3.23, SD = 1.20), support (M = 2.97, SD = 1.40) and information needs (M = 3.39, SD = 1.05) were also of great significance. Female ICs rated support options as a more important discussion topic than male ICs (*p* = 0.039, u = 0.41, Online Resource 8).

While 83.9% (*n* = 26) had discussed life threat with HCPs, only 50% (*n* = 13) found it helpful, and 16.1% (*n* = 5) wished for additional support.

### HCPs

### Perceived hope for a cure & fear of dying in patients and ICs

HCPs estimated patients as having high hope for a cure (M = 8.27, SD = 1.37, *n* = 124) and moderate fear of dying throughout their allo-HSCT journey (M = 5.33, SD = 2.22, *n* = 120), while ICs were perceived to have even higher hope (M = 8.63, SD = 1.50, *n* = 120) and greater fear (M = 6.84, SD = 2.20, *n* = 116). Significant differences were found between estimates for patients and for ICs in both hope (*p* = 0.003, u = 0,27, *n* = 120) and fear (*p* < 0.001, u = 0.062, *n* = 115, Online Resource 9a).

A weak but significant positive correlation was found between HCPs’ years of experience and perceived patient fear of dying (*r* = 0.187, *p* = 0.041, *n* = 120). Higher Death Approach Acceptance correlated with lower perceived fear in ICs (*r*=-0.235, *p* = 0.044, *n* = 74, Online Resource 9b).

### Timing when patients and ICs express a need for discussion about life threat

HCPs reported the outset of severe complications (patients: 84.8%, *n* = 106; ICs: 76.8%, *n* = 96) and the time of a change in goals of care (patients: 81.6%, *n* = 102; ICs: 75.2%, *n* = 94) as the most frequent time points when patients and ICs express a need for discussions, while the time of follow-up in the outpatient settings was perceived as less frequent (patients: 7.2%, *n* = 9; ICs: 5.6%, *n* = 7). Patients were seen as having a significantly greater need for discussions in times of complications (*p* = 0.041, Online Resource 9b).

Perceptions varied by gender, professional role, and experience, with male HCPs being more likely to report a need for discussions at diagnosis (patients: *p* = 0.038, V = 0.196; ICs: *p* = 0.025, V = 0.212). HCPs with greater experience in EOL care were more likely to report this need at diagnosis (patients: *p* = 0.036, u = 0.19) and allo-HSCT indication (patients: *p* = 0.041, u = 0.18; ICs: *p* = 0.041, u = 0.18). Additionally, HCPs who stated a need for conversations in ICs in the event of complications had, on average, cared for more EOL patients (*p* = 0.029, u = 0.20, Online Resource 9b).

Physicians were more likely than nurses and psycho-oncologists to report a need for discussions at diagnosis (patients: *p* = 0.050, V = 0.23), while psycho-oncologists most frequently faced this need at the time of indication for an allo-HSCT (*p* = 0.012, V = 0.27), on the HSCT ward (*p* = 0.025, V = 0.24) and in the outpatient setting (*p* = 0.010, V = 0.31).

Physicians reported more often than other HCPs a request for conversation in ICs at diagnosis (*p* = 0.031, V = 0.23), indication (*p* = 0.048, V = 0.22), complications (*p* = 0.007, V = 0.29), and a change in goals of care (*p* = 0.003, V = 0.30), while nurses experienced this need mainly on the ward (*p* = 0.005, V = 0.28).

HCPs with higher death acceptance were less likely to report that patients expressed a need for discussions (*p* = 0.023, u = 0.24), while those with higher escape acceptance were less likely to report this need within ICs (*p* = 0.018, u = 0.26), both at time of indication. Higher death avoidance was associated with a reduced likelihood of reporting such a need in patients in the event of severe complications (*p* = 0.034, u = 0.24).

### Preferred professional group for conversation about life threat

The majority of HCPs (97.6%, *n* = 122) considered hematologists and psycho-oncologists most suitable for discussing life threat.

Gender influenced preferences, with women being more likely to choose nurses (*p* = 0.049, φ =-0.176, Online Resource 9c) and PC specialists (*p* = 0.040, φ =-0.184), while men were less likely to select PC specialists (OR = 0.314, *p* = 0.039). Nurses saw other nurses as the most suitable discussion partner for patients and ICs, whereas physicians and psycho-oncologists were significantly less likely to choose nurses (*p* = 0.002, V = 0.29).

Age negatively correlated with choosing PC specialists (*r*=-0.202, *p* = 0.024, Online Resource 9c), confirmed by logistic regression (OR = 0.928, *p* = 0.022). Logistic regression further revealed that death avoidance was negatively associated with selecting PC specialists (OR = 0.473, *p* = 0.027, *n* = 81), while approach acceptance increased the likelihood by 93.2% (OR = 1.932, *p* = 0.049, *n* = 81).

### Comparison of the time points considered “ideal” for discussing the life threat versus the actual time points

Regarding the ideal timing to address the topic of life threat with patients and ICs, the majority of HCPs indicated that this should ideally occur at the time of a change in goals of care (79.2%), at the time of establishing the treatment indication for allo-HSCT (72.0%), or when patients themselves bring up the topic (72.8%). The diagnosis time point was also frequently considered appropriate (69.6%).

The most frequently selected time points when such conversations actually occurred in practice were changes in goals of care (68.0%) and complications (63.2%); earlier points in the care trajectory were mentioned far less frequently (Table 3).

Statistical comparison showed significant discrepancies between many ideal and actual time points. At six specific time points—diagnosis (*p* < 0.001), indication for allo-HSCT (*p* < 0.001), shortly before admission (*p* < 0.001), on the transplantation ward (*p* = 0.002), in the outpatient setting (*p* < 0.001), and at the time of a change in goals of care (*p* = 0.038), the topic was discussed significantly less often than HCPs considered ideal.

In logistic regression analyses, greater EOL-care experience significantly decreased the likelihood of considering patient-initiated discussions as the ideal time (OR = 0.967, *p* = 0.035). Furthermore, attitudes toward death significantly shaped discussion preferences, with higher escape acceptance being associated with a reduced likelihood of considering diagnosis as the ideal discussion time (OR = 0.601, *p* = 0.031), and greater death avoidance linked to a lower preference for patient-initiated discussions (OR = 0.427, *p* = 0.002). Greater fear of death was associated with a lower likelihood of a discussion during complications (OR = 0.637, *p* = 0.033) and change in goals of care (OR = 0.576, *p* = 0.023, Online Resource 11).

**Fig. 3:**
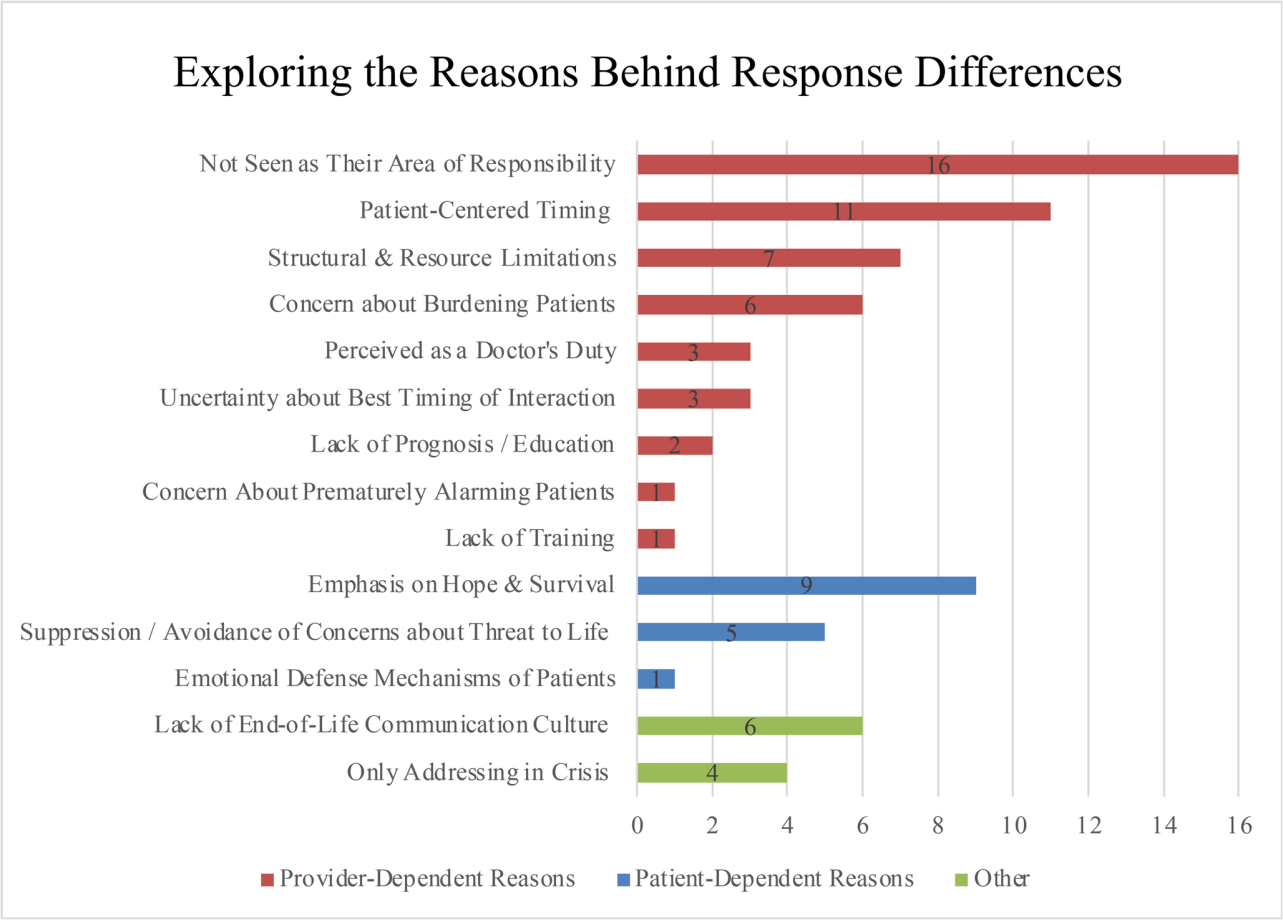
Exploring the reasons behind response differences between ideal and actual time points of conversations about life threat

Figure 3 illustrates the main reasons for discrepancies between the ideal and actual timing of conversations about life threat, based on open-ended responses by HCPs. Frequently cited provider-related factors included the perception that such conversations were not within their area of responsibility (*n* = 16), the intention to align with patient-centered timing (*n* = 11), and structural or resource-related constraints (*n* = 7).

**Table 3:** Comparison of time points being chosen as “ideally” and “actually” for a conversation about life threat by HCPs for a conversation about life threat (Total HCP sample: *n* = 125)

	Ideally discussed		Actually discussed		McNemar Test for paired responses on discussion timing
	Frequency (*n*)	Percentages %	Frequency (*n*)	Percentages %	*P*-values
Time of Diagnosis	87	69.6%	47	37.6%	*p* < 0.001
Time of Indication for allo-HSCT	90	72.0%	44	35.2%	*p* < 0.001
Shortly before admission to the transplantation ward	26	20.8%	7	5.6%	*p* < 0.001
On the transplantation ward	30	24.0%	12	9.6%	*p* = 0.002
In the outpatient clinic	22	17.6%	5	4.0%	*p* < 0.001
During the time of severe complications	90	72.0%	79	63.2%	*p* = 0.108
During a change in goals of care	99	79.2%	85	68.0%	*p* = 0.038
Patient initiates conversation	91	72.8%	82	65.6%	*p* = 0.122
No response	2	1.6%	2	1.6%	*p* = 1.000
Others	5	4.0%	6	4.8%	/

## Discussion

This study provides new insights into the communication needs of patients, ICs and HCPs regarding life threat in the context of allo-HSCT. Prognosis was rated the most important topic across patients and ICs, who reported the highest fear of dying at diagnosis and preferred early conversations with hematologists—preferences that were partly reflected in HCPs’ views. Many HCPs identified similar key moments for discussion and considered hematologists and psycho-oncologists appropriate conversation partners.

In contrast, HCPs most frequently observed patients‘ and ICs’ need for discussions about life threat during complications or when goals of care changed. Timing preferences varied significantly by professional background, gender, and experience. Attitudes toward death (e.g., avoidance, acceptance) influenced both perceived needs and actual engagement in conversations. While HCPs largely agreed.

that such discussions should ideally occur early—particularly at diagnosis, during indication setting, or at changes in goals of care—they recognized that conversations actually often take place later in the care trajectory or in reaction to complications. This highlights the importance of integrating issues related to EOL early, consistent with broader international legal frameworks that emphasize advance care planning [22, 24, 25].

Patients and ICs reported the greatest concern about life threat at the time of diagnosis, which was also when they most frequently expressed a desire to engage in conversations about this topic, a perception shared by HCPs.

However, discrepancies emerged at other time points, such as at indication, on the ward, at time of complications and at the transition from a curative to a palliative goal of care. At these stages, HCPs reported a more frequent need for conversation than patients and ICs themselves did. This suggests a mismatch between their perspectives.

These misalignments may be due to the fact that most of participating patients and ICs were in post-transplant outpatient care, and we may not have captured participants with a more unfavorable disease course and thus with likely greater existential concerns at indication, on the ward or at time of complications. Furthermore, none of the patients were in an actual PC situation, making it difficult to mentally place themselves in the reality of dying and anticipate their communication needs in such a context. Therefore, these outcomes should be interpreted cautiously and future research including patients with poorer prognosis is warranted.

The findings highlight the need for communication strategies that respect patients’ and ICs’ readiness, while meeting HCPs’ ethical duty to disclose prognosis. This is in line with Gray et al., who highlight the variability in patients’ needs regarding the depth of prognostic communication [26]. Interestingly, whereas quantitative data generally suggest a high overall demand for prognostic information [6, 27–29], qualitative studies often reveal that patients desire a more personalized approach, adapted to their unique needs, as they may feel overwhelmed by too much information [30].

Furthermore, existential concerns and communication preferences may vary by age and social context, our findings suggest that life stage may influence communication needs. Patients who self-reported having dependent children reported greater concerns about life threat after transplantation than those without. However, due to the small subgroup size, this finding should be confirmed in larger studies. Regardless of life stage, life-threat communication should be acknowledged as a process occurring within evolving mortality risk [31]. In this context, frameworks such as the SPIKES protocol may support such patient-centered, structured conversations [32].

However, communication challenges not only concern patients but also involve their immediate environment. In our study, ICs often experienced greater concerns about life threat than patients. However, only 50% of ICs who previously had conversations about life threat, found these to be helpful, suggesting unmet needs. Additionally, ICs often felt overlooked, a finding aligning with previous research [33].

This discrepancy between high concerns and limited perceived benefit of discussions suggests that existing communication practices may not sufficiently address ICs’ needs. Given these finding, communication training promoting structured triadic interactions involving patients, ICs, and HCPs may help ensure that concerns of all involved parties are acknowledged. Additional written or multimodal communication could help address these unmet needs and should be evaluated in future research.

Yet, to successfully address life threat, a suitable conversation partner is needed. Across all three study populations, hematologists were identified as the preferred professionals for such discussions. While hematologists were favored, PC specialists were also highly regarded among HCPs, with 85.6% considering them a suitable choice. In contrast, PC specialists were rated notably low by both patients (4.9%) and ICs (6.5%). These results match with studies indicating a low awareness of specialist PC in the public [34, 35]. In light of these findings, an interdisciplinary approach between hematologists and PC specialists may be beneficial not only to improve patients’ and ICs’ acceptance of PC, but also to distribute the responsibilities of communication among HCPs [36, 37].

Furthermore, a discrepancy emerged between HCPs’ preferred and actual timing of conversations about life threat: although most agreed these discussions should ideally occur early, they often take place later in the care trajectory or only in response to complications, according to HCPs‘ experience. One contributing factor to this gap may be HCPs’ individual attitudes toward death, which significantly shaped their communication behavior. In our study, greater fear of death in HCPs was associated with a lower likelihood of engaging in a discussion with patients about life threat during complications. Based on this finding, self-reflective training that addresses HCPs’ attitudes toward death may help reduce this discrepancy. Although these findings should be confirmed in greater cohorts, they align with earlier research further emphasizing the importance of self-reflective training [16, 18, 38, 39].

## Strengths and limitations

Our study shows certain limitations, namely the lack of a balanced distribution in our patient and IC population, as the majority of participants were in outpatient follow-up care. This skewed representation may affect the generalizability of our results to individuals in earlier or later (e.g. palliative) stages of treatment. In addition, the study was conducted in the German healthcare system, where transplant care structures and communication processes may differ from other systems, therefore limiting the transferability.

Furthermore, the response rate of the participants could not be determined due to strict confidentiality policies in cooperating HSCT centers, which prevented an evaluation of potential selection biases.

## Conclusion

The results of our study show divergencies in the desired communication about life threat. ICs report significant concerns but perceive insufficient support from HCPs.

Effective discussions about death require timely and patient-centered approaches that consider patients’ preferences and sociodemographic factors in order to develope meaningful and supportive conversations. Future research should explore the long-term impact of timely EOL discussions on patients’ and ICs’ concerns about life threat, as well as examine how communication needs evolve throughout the transplant trajectory in prospective longitudinal studies and develop interdisciplinary strategies to support HCPs to better approach EOL conversations.

## Supplementary Information

Below is the link to the electronic supplementary material.

Supplementary Material 1

## Data Availability

The datasets generated during and analyzed during the current study are available from the corresponding author on reasonable request.
