# Supplementary material for: How to talk about death? A cross-sectional survey on patients’, informal caregivers’ and health care professionals’ views in the setting of allogenic hematopoietic stem cell transplantation
Source: Ann Hematol. 2026 Apr 2;105(4):225. doi: 10.1007/s00277-026-06981-7 (PMC13046615; doi:10.1007/s00277-026-06981-7)
Supplement: Supplementary file 1 — Supplementary Material 1 [file 277_2026_6981_MOESM1_ESM.docx]

**Supplementary information**

Article title: How to talk about death: A cross-sectional survey on patients’, informal caregivers’ and health care professionals’ views in the setting of allogenic hematopoietic stem cell therapy

Journal: Annals of Hematology

Lisanne PREUSS, Steffen T. SIMON, Marco HERLING, Udo HOLTICK, Alinda REIMER, Berenike SCHOERGER, Sukhvir KAUR, Jithmi WELIWITAGE, Martin HELLMICH, Michael HALLEK, Carolin SCHEPERS, Johann AHN, Christof SCHEID, Roland SCHROERS, Georg-Nikolaus FRANKE, Raymond VOLTZ, Anne PRALONG

Corresponding author: Dr. Anne PRALONG, University Hospital Cologne, Kerpener Strasse 62, 50924 Cologne, Germany. Phone: +49-221-478-96538. Email: anne.pralong@uk-koeln.de

**Overview of Online Resources :**

- Online Resource 1: Survey structure for Patients, HCPs and ICs
- Online Resource 2: Self-developed questionnaire for Patients, HCPs and ICs
- Online Resource 3: Comparison of choice for conversation partner between Patients, HCPs and ICs
- Online Resource 4: Correlations and associations between ideal and actual time points chosen by the HCPs for communication about life threat and sociodemographic and profession related variables (Total HCP sample: n=125)
- Online Resource 5: Correlations between ideal and actual time points chosen by the HCPs for communication about life threat and the attitude towards death (Total HCP sample: n=125)
- Online Resource 6: Group differences in concerns about life threat between patients (n=61) and ICs (n=31)
- Online Resource 7:Associations and group differences in concerns about life threat and communication preferences by sociodemographic variables in patients (n=61)
- Online Resource 8: Associations and group differences in life threat concerns and communication preferences by sociodemographic variables in ICs (n=31)
- Online Resource 9a: Group differences in perceived hope for a cure & fear of dying in patients and ICs, reported by HCPs (n=125)
- Online Resource 9b: Associations and group differences in HCPs' perceived need for discussions with patients and ICs, by sociodemographic, profession related and psychometric variables (n=125)
- Online Resource 9c) Associations and group differences in preferred professional group by sociodemographic and profession related variables in HCPs (n=125)
- Online Resource 10: Factors influencing the timing conversation about life threat: results from logistic regression analyses
- Online Resource 11: Participating centers and recruitment overview

**Online Resource 1: Survey structure for Patients, ICs and HCPs**

**Patients’ survey**

| **Development type** | **Question type** | | **Number of items** | **Dimensions** | |
| --- | --- | --- | --- | --- | --- |
| **Thoughts about life threat** | | | | | |
| Self-developed | - 5-point Likert scale (0=not at all to 5=very much) | 7 (see online resource 2 for item details) | | Situations in which a conversation about life threat should take place | |
| **Communication about life threat** | | | | | |
| Self-developed | - 5-point Likert scale (1=not at all to 5=very much) - Multiple choice - Single choice - Open ended questions | 6 (see online resource 2 for item details) | | What is the setting, time point and preferred discussion partners for conversation about life threat? What do patients wish for in this type of conversation and do they prefer more support? | |
| **Personal attitudes toward death** | | | | | |
| **Death acceptance subscale of the *Life Attitude Profile-Revised* (LAP-R)** (16,17) | | | | | |
| Standardized, validated | - 7-point Likert scale (1=strongly disagree to 7=strongly agree) | | 8 (see online resource 2 for item details) | | **-** |
| **Demographics and medical characteristics** | | | | | |
| Age, mother tongue, gender, religion, family status, living situation, level of education, as well as medical data such as: time of transplantation, underlying hematological disease, donor type, HCT-CI, Transplant-associated complications | | | | | |

**Informal Caregivers’ survey**

| **Development type** | **Question type** | | **Number of items** | **Dimensions** | |
| --- | --- | --- | --- | --- | --- |
| **Thoughts about life threat** | | | | | |
| Self-developed | - 5-point Likert scale (0=not at all to 5=very much) | 7 (see online resource 2 for item details) | | Situations in which a conversation about life threat should take place | |
| **Conversation about life threat with the patient** | | | | | |
| Self-developed | - 5-point Likert scale (1=not at all to 5=very much) - Multiple choice - Single choice - Open ended questions | 8 (see online resource 2 for item details) | | What is the setting and time point for conversation about life threat with the patient?  What are the hopes and fears connected with a conversation about life threat? | |
| **Conversation about life threat with the HCP** | | | | | |
| Self-developed | - 5-point Likert scale (1=not at all to 5=very much) - Multiple choice - Single choice - Open ended questions | 6 (see online resource 2 for item details) | | What is the setting, time points and preferred discussion partners for conversation about life threat with the HCPs? What do ICs wish for in this type of conversation and do they prefer more support? | |
| **Personal attitudes toward death** | | | | | |
| **Death acceptance subscale of the *Life Attitude Profile-Revised* (LAP-R)** (16,17) | | | | | |
| Standardized, validated | 7-point Likert scale (1=strongly disagree to 7=strongly agree) | | 8 | | **-** |
| **Demographics and medical characteristics** | | | | | |
| Age, mother tongue, gender, religion, family status, living situation, level of education, as well as medical data such as: time of transplantation, underlying hematological disease, donor type, HCT-CI, Transplant-associated complications | | | | | |

**HCPs’ survey**

| **Development type** | **Question type** | | **Number of items** | **Dimensions** | | |
| --- | --- | --- | --- | --- | --- | --- |
| **HCPs’ assessment of hope for a cure and fear of dying in patients and ICs** | | | | | | |
| Self-developed | 10-point Likert scale (0=not present at all to 10=extremely pronounced) | 4 (see online resource 2 for item details) | | Hope for a cure in patients and ICs, as well as fear of dying in patients and ICs | | |
| **HCPs’ assessment of Patients’ and ICs’ need for discussion** | | | | | | |
| Self-developed | Multiple choice | 3 (online resource 2 for item details) | | 1. Situations in which patients express a need for discussion about life threat and fear of dying 2. Situations in which ICs express a need for discussion about life threat and fear of dying 3. Professional groups that should offer discussion about life threat and fear of dying | | |
| **At which time point HCPs address Life Threat / Fear of dying** | | | | | | |
| Self-developed | Multiple choice | 2 (see online resource 2 for item details) | | 1.Ideal time points for addressing the topic of life threat and fear of dying, regardless of patients’ or informal caregivers’ preference  2. Actual time points for addressing the topic of life threat and fear of dying, regardless of patients’ or informal caregivers’ preferences | | |
| **Personal attitudes toward death** | | | | | | |
| ***Death Attitude Profile-Revised* (DAP-R)** (18,19) | | | | | | |
| Standardized, validated | 7-point Likert scale (1=strongly disagree to 7=strongly agree) | | 32 | | Fear of death: Negative thoughts and feelings about death and dying  Death avoidance: Avoiding thoughts about death to reduce death anxiety  Neutral acceptance: View of death as something that is accepted, neither feared nor welcomed  Approach acceptance: View of death as a gateway to a positive afterlife  Escape acceptance: View of death as an escape from a painful existence | |
| **Death acceptance subscale of the *Life Attitude Profile-Revised* (LAP-R)** (16,17) | | | | | | |
| Standardized, validated | 7-point Likert scale (1=strongly disagree to 7=strongly agree) | | 8 | | **-** |  |
| **Demographics and clinical characteristics** | | | | | | |
| Age, mother tongue, gender, profession, years of clinical practice with allo-HSCT patients, annual number of allo-HSCT patients and annual number of patients cared for during their dying phase | | | | | | |

**Online Resource 2: Self-developed questionnaire for Patients, ICs and HCPs**

**Patients:**

## *Thoughts on Life Threat*

Please indicate how strongly thoughts about life threat and the fear of dying have occupied you during your illness. If the described situation has (not yet) occurred or does not apply, please check "Does not apply."

| I was preoccupied with concerns about life threat and fear of dying… | not at all | a little | mode-rately | quite a lot | very much | does not apply |
| --- | --- | --- | --- | --- | --- | --- |
| 1. ... when my cancer was diagnosed. | 🔿 | 🔿 | 🔿 | 🔿 | 🔿 | 🔿 |
| 2. ... when the first cancer treatment started, and during the first weeks or months of treatment. | 🔿 | 🔿 | 🔿 | 🔿 | 🔿 | 🔿 |
| 3. ... when doctors recommended an allogeneic stem cell transplantation to me (indication). | 🔿 | 🔿 | 🔿 | 🔿 | 🔿 | 🔿 |
| 4. ... when I knew I would be admitted to the transplantation ward in a few days. | 🔿 | 🔿 | 🔿 | 🔿 | 🔿 | 🔿 |
| 5. ... while I was on the transplantation ward. | 🔿 | 🔿 | 🔿 | 🔿 | 🔿 | 🔿 |
| 6. ... after being on the transplantation ward. | 🔿 | 🔿 | 🔿 | 🔿 | 🔿 | 🔿 |
| 7. ... in a situation where there was no hope of a cure. | 🔿 | 🔿 | 🔿 | 🔿 | 🔿 | 🔿 |

## *Conversations About Life Threat*

We would like to learn more about your expectations for a conversation about life threat and the fear of dying due to your cancer diagnosis.

1. Should a family member or loved one be present during a conversation about life threat

🔿 Yes

🔿No

🔿 Don’t know

2. With whom should a conversation about life threat take place?

🔿 Oncologist

🔿 General practitioner

🔿 Nurse/Care coordinator

🔿 Chaplain/Spiritual advisor

🔿 Palliative care specialist

🔿 Psychologist

🔿 Volunteer

🔿 Other persons, namely: __________________________

3. When should a conversation about life threat take place?

🔿 At the time of my cancer diagnosis

🔿 At the start of the first cancer treatment and during the first weeks or months of treatment

🔿 When an allogeneic stem cell transplantation is recommended (indication)

🔿 Shortly before admission to the transplantation ward

🔿 While on the transplantation ward

🔿 After being on the transplantation ward

🔿 In a situation where there was no hope for a cure

🔿 Another time, namely: __________________________

4. How important are the following aspects in a conversation about life threat?

|  | not at all | a little | mode  -rately | quite a lot | very much |
| --- | --- | --- | --- | --- | --- |
| Prognosis (chance of a cure and risk of dying) | 🔿 | 🔿 | 🔿 | 🔿 | 🔿 |
| Coping (What helps me in this situation?) | 🔿 | 🔿 | 🔿 | 🔿 | 🔿 |
| Support (What services are available?) | 🔿 | 🔿 | 🔿 | 🔿 | 🔿 |
| Information needs (Who/what can I ask?) | 🔿 | 🔿 | 🔿 | 🔿 | 🔿 |

5. If you have already had a conversation about life threat, did you find it helpful?

🔿Yes

If yes, what was helpful? ______________________________________________

🔿 No

If no, why not? ______________________________________________________

6. Would you like additional support on the topic of life threat and the fear of dying due to your cancer diagnosis, beyond just a conversation?

🔿 Yes

🔿 No

If yes, what kind of support? __________________________________________

**Informal Caregivers:**

## *Thoughts on Life Threat*

Please indicate at which point in the illness of your relative you were strongly occupied with the thoughts of life threat and fear of them dying. (If the described situation has (not yet) occurred or does not apply, please check "Does not apply.")

| I was preoccupied with concerns about life threat and fear of my relative dying… | Not at all | A little | Moderately | Quite a lot | Very much | Does not apply |
| --- | --- | --- | --- | --- | --- | --- |
| 1. … when the cancer was diagnosed. | 🔿 | 🔿 | 🔿 | 🔿 | 🔿 | 🔿 |
| 2. … when the first cancer therapy started and during the first weeks or months of treatment. | 🔿 | 🔿 | 🔿 | 🔿 | 🔿 | 🔿 |
| 3. … when doctors recommended an allogeneic stem cell transplantation (indication). | 🔿 | 🔿 | 🔿 | 🔿 | 🔿 | 🔿 |
| 4. … when I knew that my relative would be admitted to the transplantation ward in a few days. | 🔿 | 🔿 | 🔿 | 🔿 | 🔿 | 🔿 |
| 5. … when my relative was on the transplantation ward. | 🔿 | 🔿 | 🔿 | 🔿 | 🔿 | 🔿 |
| 6. … after my relative was on the transplantation ward. | 🔿 | 🔿 | 🔿 | 🔿 | 🔿 | 🔿 |
| 7. … in a situation where there was no hope for a cure. | 🔿 | 🔿 | 🔿 | 🔿 | 🔿 | 🔿 |

## *Conversations About Life Threat with your Relative*

We would like to learn more about your expectations for a conversation about life and the fear of your relative dying.

| 1. How often do you talk with your relative about the life-threatening nature of their illness? |
| --- |
| 🔿 Never  🔿 Rarely  🔿 Sometimes  🔿 Often  🔿 Very often |
| 2. Have you explicitly talked about their final phase of life, possible death, and dying? |
| 🔿 Yes (continue with 3)  🔿No (continue with 4)  🔿Don’t know |
| 3. If Yes: What was the reason to talk about the final phase of life (remaining days)? |
| _______________________________________________________________ |
| 4. If No: Why not? |
| _______________________________________________________________ |
| Do you wish to discuss topics like dying and death with your relative?  🔿 Yes  🔿 No  🔿 Don’t know |

## *Conversations About Life Threat with the Treatment Team*

We would like to learn more about your expectations for a conversation with the treatment team about life threat and the fear of your relative dying.

| 1. Should a conversation about life threat take place with you alone (without your relative)? |
| --- |
| 🔿 Yes  🔿 No  🔿 Don’t know |
| 2. With whom should a conversation about life threat take place? |
| 🔿 Oncologist  🔿 General practitioner  🔿 Nurse/Care coordinator  🔿 Chaplain/Spiritual advisor  🔿 Palliative care specialist  🔿 Psychologist  🔿 Volunteer  🔿 Other: _____________ |
| 3. At what point should a conversation with the treatment team about life threat take place? |
| 🔿At diagnosis  🔿At the start of the first cancer therapy  🔿When a stem cell transplant is recommended  🔿Shortly before the transplantation ward stay  🔿 During the stay on the transplantation ward  🔿 After the transplantation ward stay  🔿 When no hope for a cure exists  🔿 Other: ____________ |

**HCPs**

## *Extent of Hope for a Cure and Fear of Dying*

Think about your patients undergoing an allogeneic stem cell transplantation (allo-SCT), who have a realistic chance of a cure AND a significant risk of dying (30 to 70%), as well as their relatives.

0 = not present at all; 10 = extremely pronounced

|  | 0 | 1 | 2 | 3 | 4 | 5 | 6 | 7 | 8 | 9 | 10 |
| --- | --- | --- | --- | --- | --- | --- | --- | --- | --- | --- | --- |
| 1. How pronounced is the hope for a cure among your patients in general? | 🔿 | 🔿 | 🔿 | 🔿 | 🔿 | 🔿 | 🔿 | 🔿 | 🔿 | 🔿 | 🔿 |
| 2. How pronounced is the fear of dying among your patients in general? | 🔿 | 🔿 | 🔿 | 🔿 | 🔿 | 🔿 | 🔿 | 🔿 | 🔿 | 🔿 | 🔿 |
| 3. How pronounced is the hope for a cure of the patients among their relatives in general? | 🔿 | 🔿 | 🔿 | 🔿 | 🔿 | 🔿 | 🔿 | 🔿 | 🔿 | 🔿 | 🔿 |
| 4. How pronounced is the fear of the patient’s death among their relatives in general? | 🔿 | 🔿 | 🔿 | 🔿 | 🔿 | 🔿 | 🔿 | 🔿 | 🔿 | 🔿 | 🔿 |

## *Discussion Needs of Patients*

When do your patients undergoing an allo-SCT express a need for discussions about life threat or the fear of dying? (Multiple answers possible)

🔿 At the diagnosis of cancer

🔿 When the indication for a first allo-SCT is given

🔿 Shortly before admission to the transplantation ward

🔿 On the transplantation ward

🔿 In the outpatient clinic within 12 months after the allo-SCT

🔿 In case of severe, prognostically relevant complications

🔿 When the treatment goal changes (from curative to palliative)

🔿 None of these / Cannot answer

🔿 Other, namely: ______________________

## *Discussion Needs of Relatives*

When do the relatives of patients undergoing an allo-SCT express a need for discussions about life threat or the fear of the patient dying? (Multiple answers possible)

🔿 At the diagnosis of cancer

🔿 When the indication for a first allo-SCT is given

🔿 Shortly before admission to the transplantation ward

🔿 On the transplantation ward

🔿 In the outpatient clinic within 12 months after the allo-SCT

🔿 In case of severe, prognostically relevant complications

🔿 When the treatment goal changes (from curative to palliative)

🔿 None of these / Cannot answer

🔿 Other, namely: ______________________

## *Conversation Partners for Discussions about Life Threat / Fear of Dying*

Which professional groups, in your opinion, should offer conversations about life threat / fear of dying for patients undergoing an allo-SCT and their relatives? (Multiple answers possible)

| 🔿 Treating hematologist |
| --- |
| 🔿 Psycho-oncologist |
| 🔿 Nursing staff |
| 🔿 Guides/Coordinators |
| 🔿 General practitioner |
| 🔿 Palliative care physician |
| 🔿 Chaplain/Spiritual advisor |
| 🔿 Volunteers |
| 🔿 None of these / Cannot answer |
| 🔿 Other, namely: ______________________ |

## *Addressing Life Threat / Fear of Dying*

a) At which points in time should the topic "life threat / fear of dying" ideally be addressed with patients undergoing an allo-SCT and their relatives, irrespective of their wishes? (Multiple answers possible)

🔿 At the diagnosis of cancer

🔿 When the indication for a first allo-SCT is given

🔿 Shortly before admission to the transplantation ward

🔿 On the transplantation ward

🔿 In the outpatient clinic within 12 months after the allo-SCT

🔿 In case of severe, prognostically relevant complications

🔿 When the treatment goal changes (from curative to palliative)

🔿 None of these / Cannot answer

🔿 Other, namely: ______________________

b) At which points in time do you actually address the topic "life threat / fear of dying" with patients undergoing an allo-SCT and their relatives, irrespective of their wishes? (Multiple answers possible)

🔿 At the diagnosis of cancer

🔿 When the indication for a first allo-SCT is given

🔿 Shortly before admission to the transplantation ward

🔿 On the transplantation ward

🔿 In the outpatient clinic within 12 months after the allo-SCT

🔿 In case of severe, prognostically relevant complications

🔿 When the treatment goal changes (from curative to palliative)

🔿 None of these / Cannot answer

🔿 Other, namely: ______________________

## *Discrepancies Between B5 and B6*

If you selected different answers in the last two questions (B5 a and B6 b): What do you think is the reason for this?

**Online Resource 3: Comparison of choice for conversation partner between Patients, HCPs and ICs**

|  | Patients (n=61) | | ICs (n=31) | | HCPs (n=125) | |
| --- | --- | --- | --- | --- | --- | --- |
|  | Frequencies (n) | Percentages % | Frequencies (n) | Percentages % | Frequencies (n) | Percentages % |
| Hemato-oncologist | 47 | 77.0% | 21 | 67.7% | 122 | 97.6% |
| Psychologist / Psycho-oncologist | 22 | 36.1% | 6 | 19.4% | 122 | 97.6% |
| Nurses | 2 | 3.3% | 3 | 9.7% | 98 | 78.4% |
| Guides/Coordinators |  |  |  |  | 19 | 15.2% |
| General practicioner | 6 | 9.8% | 3 | 9.7% | 42 | 33.6% |
| Palliative care physician | 3 | 4.9% | 2 | 6.5% | 107 | 85.6% |
| Spiritual counselor | 5 | 8.2% | 2 | 6.5% | 94 | 75.2% |
| Volunteers | 0 | 0.0% | 0 | 0.0% | 30 | 24.0% |
| No response | 0 | 0.0% | 0 | 0.0% | 0 | 0.0% |
| others | 9 | 14.8% | 3 | 9.75 | 1 | 0.8% |

| **Online Resource 4:** **Correlations and associations between ideal and actual time points chosen by the HCPs for communication about life threat and sociodemographic and profession-related variables (Total HCP sample: n=125)** | | | | | | | | | | | | | | |
| --- | --- | --- | --- | --- | --- | --- | --- | --- | --- | --- | --- | --- | --- | --- |
| Life threat ideally discussed | | | | | | | | Life threat actually discussed | | | | | | |
| Chosen time point | Frequency of yes: n (%), | Gender | Age | Years of work experience | Number of allo-HSCT patients cared for | Number of supported patients in dying phase | Professional group  (Exact Fisher Test) | Frequency of yes: n (%), | Gender | Age | Years of work experience | Number of allo-HSCT patients cared for | Number of supported patients in dying phase | professional group  (Exact Fisher Test) |
| Time of Diagnosis | 87 (69.6%) | **Φ=0.281, p=0.002*** | r=-0.180, p=0.045* | r=-0.227  p=0.011* | r=0.005  p=0.954 | r=0.006  p=0.946 | Phy>Nur (p=0.003)* | 47 (37.6%) | **Φ=0.290, p=0.001*** | r=-0.124  p=0.168 | r=-0.257  p=0.004* | r=0.005  p=0.958 | r=-0.035  p=0.701 | Phy & Psy>Nur (p<0.001)* |
| Time of Indication for allo-HSCT | 90 (72.0%) | **Φ=0.209, p=0.020*** | r=0.054 p=0.550 | r=-0.087  p=0.336 | r=0.043  p=0.631 | r=-0.028  p=0.758 | Phy>Nur (p=0.003)* | 44 (35.2%) | **Φ=0.163, p=0.068** | r=0.086  p=0.340 | r=-0.016  p=0.859 | r=0.118  p=0.189 | r=0.001  p=0.993 | Phy & Psy>Nur (p<0.001)* |
| Shortly before admission to the transplantation ward | 26 (20.8%) | **Φ=0.073, p=0.414** | r=-0.125, p=0.166 | r=-0.101  p=0.262 | r=-0.011  p=0.906 | r=0.085  p=0.348 | p=0.738 | 7 (5.6%) | **Φ=0.018, p=0.838** | r=0.143  p=0.111 | r=0.101  p=0.261 | r=0.164  p=0.067 | r=0.123  p=0.173 | p=0.374 |
| On the transplantation ward | 30 (24.0%) | **Φ=0-048, p=0.595** | r=-0.158 p=0.078 | r=-0.062  p=0.491 | r=-0.151  p=0.093 | r=0.016  p=0.862 | p=0.946 | 12 (9.6%) | **Φ=-0.039, p=0.662** | r=0.011  p=0.905 | r=0.010  p=0.913 | r=0.062  p=0.492 | r=0.046  p=0.611 | p=0.431 |
| In the outpatient clinic | 22 (17.6%) | **Φ=0.145, p=0.104** | r=-0.111, p=0.219 | r=-0.063 p=0.485 | r=-0.075  p=0.406 | r=-0.021  p=0.817 | p=0.250 | 5 (4.0%) | **Φ=0.087, p=0.331** | r=0.042  p=0.643 | r=0.088  p=0.328 | r=-0.010  p=0.915 | r=-0.007  p=0.937 | p=1.000 |
| During time of complications | 90 (72.0%) | **Φ=-0.047, p=0.601** | r=-0.192, p=0.032* | r=-0.041 p=0.652 | r=-0.055  p=0.542 | r=0.030  p=0.741 | p=0.863 | 79 (63.2%) | **Φ=0.137, p=0.126** | r=0.031  p=0.732 | r=0.104  p=0.246 | r=0.045  p=0.621 | r=0.057  p=0.530 | Phy>Nur &Psy (p=0.003)* |
| During Therapy Goal changes (curative to palliative) | 99 (79.2%) | **Φ=0.21p=0.019*** | r=-0.161, p=0.073 | r=-0.156  p=0.082 | r=-0.145  p=0.107 | r=-0.037  p=0.682 | Phy>Nur (p=0.017)* | 85 (68.0%) | **Φ=0.129, p=0.148** | r=0.063  p=0.486 | r=0.108  p=0.228 | r=0.208  p=0.020* | r=0.166  p=0.064 | Phy>Nur  & Psy  (p=0.003)* |
| Patient initiates conversation | 91 (72.8%) | **Φ=0.049, p=0.585** | r=-0.130, p=0.148 | r=-0.174  p=0.052 | r=-0.182  p=0.043* | r=-0.200  p=0.026* | p=0.112 | 82 (65.6%) | **Φ=-0.143, p=0.110** | r=-0.001  p=0.988 | r=-0.065  p=0.473 | r=-0.084  p=0.353 | r=-0.099  p=0.271 | p=0.675 |
| No response | 2 (1.6%) | **Φ=-0.102, p=0.252** | r=0.016, p=0.863 | r=0.140, p=0.120 | r=-0.053  p=0.559 | r=-0.072  p=0.426 | p=0.566 | 2 (1.6%) | **Φ=0.159, p=0.076** | r=0.023  p=0.798 | r=0.019  p=0.834 | r=-0.018  p=0.845 | r=-0.053  p=0.561 | p=0.566 |
| Others | 5 (4.0%) | **Φ=-0.080, p=0.369** | r=-0.060, p=0.507 | r=-0.047  p=0.599 | r=-0.047  p=0.602 | r=-0.001  p=0.991 | p=0.250 | 6 (4.8%) | **Φ=-0.080, p=0.369** | r=-0.075  p=0.407 | r=-0.101  p=0.263 | r=-0.021  p=0.817 | r=-0.059  p=0.516 | p=0.498 |
| **Φ:** Phi-Coefficient, r: Point-Biserial Pearson Correlation, *p≤0.05 | | | | | | | | | | | | | | |

**Online Resource 5: Correlations between ideal and actual time points chosen by the HCPs for communication about life threat and the attitude towards death (Total HCP sample: n=125)**

| Chosen time point: life threat ideally discussed | | | | | | | | Chosen time point: life threat actually discussed | | | | | | |
| --- | --- | --- | --- | --- | --- | --- | --- | --- | --- | --- | --- | --- | --- | --- |
|  | Frequency of yes: n (%) | LAP-R | Fear of the Death (DAP-R) | Death Avoidance (DAP-R) | Neutral acceptance (DAP-R) | Approach acceptance (DAP-R) | Escape acceptance (DAP-R) | Frequency of yes: n (%) | LAP-R | Fear of the Death (DAP-R) | Death Avoidance (DAP-R) | Neutral acceptance (DAP-R) | Approach acceptance (DAP-R) | Escape acceptance (DAP-R) |
| Time of Diagnosis | 87 (69.6%) | r=0.013  p=0.898 | r=-0.141  p=0.208 | r=-0.167  p=0.137 | r=-0.039  p=0.728 | r=-0.195  p=0.080 | r=-0.225  p=0.044* | 47 (37.6%) | r=-0.033  p=0.740 | r=-0.158  p=0.160 | r=-0.065  p=0.564 | r=-0.003  p=0.980 | r=-0.218  p=0.050* | r=-0.182  p=0.104 |
| Time of Indication for allo-HSCT | 90 (72.0%) | r=-0.048  p=0.630 | r=-0.118  p=0.292 | r=-0.085  p=0.451 | r=-0.038  p=0.733 | r=-0.100  p=0.373 | r=-0.078  p=0.487 | 44 (35.2%) | r=-0.061  p=0.543 | r=-0.043  p=0.704 | r=-0.036  p=0.748 | r=-0.118  p=0.294 | r=-0.058  p=0.606 | r=-0.015  p=0.987 |
| Shortly before admission to the transplantation ward | 26 (20.8) | r=0.020  p=0.842 | r=-0.007  p=0.952 | r=0.055  p=0.625 | r=0.099  p=0.380 | r=0.003  p=0.978 | r=-0.097  p=0.389 | 7 (5.6%) | r=-0.258  p=0.008* | r=0.187  p=0.094 | r=0.049  p=0.663 | r=-0.004  p=0.972 | r=0.048  p=0.671 | r=0.060  p=0.594 |
| On the transplantation ward | 30 (24.0%) | r=0.053  p=0.597 | r=-0.066  p=0.557 | r=-0.074  p=0.512 | r=0.043  p=0.702 | r=-0.015  p=0.896 | r=-0.189  p=0.091 | 12 (9.6%) | r=0.124  p=0.212 | r=0.002  p=0.989 | r=0.175  p=0.117 | r=0.069  p=0.538 | r=0.132  p=0.239 | r=0.033  p=0.769 |
| In the outpatient clinic | 22 (17.6%) | r=0.114  p=0.253 | r=-0.054  p=0.631 | r=-0.070  p=0.534 | r=0.062  p=0.580 | r=0.003  p=0.978 | r=-0.011  p=0.920 | 5 (4.0%) | r=0.025  p=0.804 | r=-0.015  p=0.894 | r=0.136  p=0.225 | r=-0.055  p=0.625 | r=-0.103  p=0.359 | r=-0.054  p=0.631 |
| During time of complications | 90 (72.0%) | r=0.018  p=0.860 | r=-0.104  p=0.357 | r=-0.115  p=0.305 | r=-0.107  p=0.343 | r=0.034  p=0.766 | r=-0.063  p=0.576 | 79 (63.2%) | r=0.107  p=0.283 | r=-0.220  p=0.048 | r=-0.035  p=0.757 | r=0.034  p=0.766 | r=-0.016  p=0.885 | r=-0.168  p=0.134 |
| During Therapy Goal changes (curative to palliative) | 99 (79.2%) | r=0.038  p=0.703 | r=-0.064  p=0.572 | r=-0.136  p=0.226 | r=-0.058  p=0.605 | r=0.034  p=0.764 | r=-0.010  p=0.932 | 85 (68.0%) | r=0.072  p=0.467 | r=-0.263  p=0.018 | r=-0.077  p=0.496 | r=0.015  p=0.893 | r=-0.112  p=0.320 | r=0.007  p=0.953 |
| Patient initiates conversation | 91 (72.8%) | r=-0.071  p=0.477 | r=-0.063  p=0.563 | r=-0.345  p=0.002* | r=-0.126  p=0.261 | r=0.004  p=0.975 | r=0.084  p=0.455 | 82 (65.6%) | r=0.006  p=0.954 | r=-0.131  p=0.245 | r=-0.240  p=0.031* | r=0.031  p=0.787 | r=0.023  p=0.842 | r=0.109  p=0.333 |
| No response | 2 (1.6%) | r=-0.024  p=0.803 | r=0.072  p=0.523 | r=0.138  p=0.219 | r=-0.102  p=0.366 | r=0.102  p=0.365 | r=0.049  p=0.664 | 2 (1.6%) | r=-0.115  p=0.247 | r=0.123  p=0.275 | r=-0.146  p=0.192 | r=-0.016  p=0.888 | r=-0.075  p=0.507 | r=-0.027  p=0.814 |
| Others | 5 (4.0%) | r=0.015  p=0.883 | r=-0.045  p=0.693 | r=-0.031  p=0.782 | r=0.006  p=0.961 | r=0.057  p=0.015 | r=0.009  p=0.939 | 6 (4.8%) | r=0.030  p=0.766 | r=0.052  p=0.645 | r=-0.043  p=0.702 | r=-0.045  p=0.692 | r=0.061  p=0.586 | r=0.064  p=0.572 |
| r: Point-Biserial Pearson Correlation, *p≤0.05 | | | | | | | | | | | | | | |

**Online Resource 6: Group differences in concerns about life threat between patients (n=61) and ICs (n=31)**

| Comparison | Test | Result | n | Interpretation |
| --- | --- | --- | --- | --- |
| Patients’ vs. ICs’ concerns about life threat at diagnosis | Mann-Whitney-U | p = 0.019*, u=0.25 | 59 Pat. 30 ICs | ICs reported higher concerns than patients |
| Concerns about life threat at start of first cancer therapy | Mann-Whitney-U | p=0.047*, u=0.22 | 56 Pat. 29 ICs | ICs reported higher concerns than patients |
| Concerns about life threat at indication for allo-HSCT | Mann-Whitney-U | p=0.083, u=0.18 | 60 Pat. 30 ICs | No significant difference |
| Concerns about life threat shortly before admission to transplantation ward | Mann-Whitney-U | p=0.003*, u=0.32 | 57 Pat. 30 ICs | ICs reported higher concerns than patients |
| Concerns about life threat on transplantation ward | Mann-Whitney-U | p=0.021*, u=0.26 | 54 Pat. 28 ICs | ICs reported higher concerns than patients |
| Concerns about life threat after transplantation ward | Mann-Whitney-U | p=0.054, u=0.23 | 45 Pat. 28 ICs | No significant difference |
| Concerns about life threat in a situation where there was no hope for a cure | Mann-Whitney-U | p=0.052, u=0.43 | 13 Pat. 12 ICs | No significant difference |
| u=effect size calculated as Z/√N, *p≤0.05 | | | | |

**Online Resource 7:Associations and group differences in concerns about life threat and communication preferences by sociodemographic variables in patients (n=61)**

| Variable | Outcome | Test | Result | n | Interpretation |
| --- | --- | --- | --- | --- | --- |
| Dependent children | Concerns about life threat after transplantation ward | Mann-Whitney-U | p=0.038, u=0.32 | 44 (10 with, 34 without dependent children, definded as | Patients who self-reported having dependent children reported higher concerns after transplantation compared to those without |
| Age | Psychologist preference | Point-biserial correlation | r=−0.321, p=0.012* | 61 | Younger patients more frequently preferred psychologists as discussion partners |
| Age | Nurse preference | Point-biserial correlation | r=−0.290, p=0.023* | 61 | Younger patients more frequently preferred nurses as discussion partners |
| Marital status | Hematologist preference | Chi-square (Fisher’s exact test) | p=0.023*, V=0.369 | 60 | Married/partnered patients more frequently preferred hematologists as discussion partners |
| Marital status | Spiritual counselor preference | Chi-square (Fisher’s exact test) | p=0.004*, V=0.444 | 60 | Unmarried, widowed or divorced patients more frequently preferred spiritual counselors as discussion partners |
| Treatment timing | Spiritual counselor preference | Chi-square (Fisher’s exact test) | p=0.021*, V=0.373 | 61 | Patients in inpatient care more frequently preferred spiritual counselors as discussion partners than those in outpatient follow-up ^a^ |
| Marital status | Preferred timing of discussion | Chi-square (Fisher’s exact test) | p=0.015*, V=0.448 | 59 | Married patients more frequently preferred discussion at diagnosis |
| Religiosity | Preferred timing of discussion | Chi-square (Fisher’s exact test) | p=0.032*, V=0.434 | 59 | Non-religious patients more frequently preferred later time points |
| Gender | Information needs as discussion topic | Mann-Whitney-U | p=0.025*, r=0.286 | 61 | Female patients rated information needs as more important than male patients |
| ^a^ interpret with caution given the small subgroup sizes V = Cramér's V; r = effect size (point-biserial correlation); u=effect size calculated as Z/√N, n reflects valid responses excluding 'not applicable', *p≤0.05 | | | | | |

**Online Resource 8: Associations and group differences in life threat concerns and communication preferences by sociodemographic variables in ICs (n=31)**

| Variable | Outcome | Test | Result | n | Interpretation |
| --- | --- | --- | --- | --- | --- |
| Age | Concerns about life threat in a situation where there was no hope for a cure | Spearman correlation | ρ=−0.594, p=0.042* | 12 | Younger ICs reported higher concerns in a hypothetical palliative situation |
| Gender | Concerns about life threat on transplantation ward | Mann-Whitney-U | p=0.049*, u=0.39 | 28 | Female ICs reported higher concerns on transplantation ward than male ICs^a^ |
| Gender | Concerns about life threat after transplantation ward | Mann-Whitney-U | p=0.013*, u=0.44 | 28 | Female ICs reported higher concerns after transplantation than male ICs |
| Gender | Concerns about life threat in a situation where there was no hope for a cure | Mann-Whitney-U | p=0.009*, u=0.95 | 12 | Female ICs reported higher concerns in a hypothetical palliative situation than male ICs^a^ |
| Age | Feeling that discussing life threat would take away hope from their relative | Kruskal-Wallis | p= 0.038*, η²=0.16 | 31 | Older ICs more often felt that discussing life threat would take away hope from their relative than younger ICs |
| Gender | Importance of discussing support in a conversation about life threat | Mann-Whitney-U | p= 0.039*, u=0.41 | 31 | Female ICs rated support options as a more important topic in conversations about life threat than male ICs |
| ^a^ interpret with caution: small sample size may inflate effect size, u=effect size calculated as Z/√N; ρ=Spearman's rank correlation coefficient, η² ( small ≥ 0.01, medium ≥ 0.06, large ≥ 0.14), *p≤0.05 | | | | | |

**Online Resource 9a: Group differences in perceived hope for a cure & fear of dying in patients and ICs, reported by HCPs (n=125)**

| Comparison | Test | Result | n | Interpretation |
| --- | --- | --- | --- | --- |
| Patients' vs. ICs' perceived hope for a cure | Wilcoxon | p=0.003*, u=0.27 | n=120 | ICs were perceived to have higher hope than patients |
| Patients' vs. ICs' perceived fear of dying | Wilxocon | p<0.001*, u=0.062 | n=115 | ICs were perceived to have higher fear of the patient’s death, than patients themselves |
| effect sizes were calculated as u= Z/√N, *p≤0.05 | | | | |

**Online Resource 9b: Associations and group differences in HCPs' perceived need for discussions with patients and ICs, by sociodemographic, profession related and psychometric variables (n=125)**

| Variable | Outcome | Test | Result | n | Interpretation |
| --- | --- | --- | --- | --- | --- |
| Patients‘ vs. ICs‘ perceived need for discussions at times of complication | - | McNemar | p=0.041, g=0.25 | 125 | Patients were seen as having a significantly greater need for discussions in times of complications compared to ICs |
| HCPs' years of experience | Perceived fear of dying in patients | Spearman correlation | ρ=0.187, p = 0.041* | 120 | More experienced HCPs reported a higher perceived fear of dying in patients |
| Death Approach Acceptance | Perceived fear of the patient’s death in ICs | Spearman correlation | ρ=−0.235, p = 0.044* | 74 | Higher acceptance of death correlated with lower perceived fear |
| Gender | Perceived need for discussions at diagosis in patients | Chi-square (Fisher’s exact test) | p=0.038*, V=0.196 | 125 | Male HCPs being more likely to report a need for discussions at diagnosis in patients |
| Gender | Perceived need for discussions at diagosis in ICs | Chi-square (Fisher’s exact test) | p=0.025*, V=0.212 | 125 | Male HCPs being more likely to report a need for discussions at diagnosis in ICs |
| Experience in EOL care | Perceived need for discussions at diagosis in patients | Mann-Whitney-U | p=0.036*, u=0.19 | 125 | HCPs with greater experience in EOL care were more likely to report a need for discussion at diagnosis in patients |
| Experience in EOL care | Perceived need for discussions at allo-HSCT indication in patients | Mann-Whitney-U | p=0.041*, u=0.18 | 125 | HCPs with greater experience in EOL care were more likely to report a need for discussion at indication for allo-HSCT in patients |
| Experience in EOL care | Perceived need for discussions at allo-HSCT indication in ICs | Mann-Whitney-U | p=0.041*, u=0.18 | 125 | HCPs with greater experience in EOL care were more likely to report a need for discussion at diagnosis in ICs |
| Experience in EOL care | Perceived need for discussions at allo-HSCT indication in ICs | Mann-Whitney-U | p=0.029*, u=0.20 | 125 | HCPs with greater experience in EOL care were more likely to report a need for discussion at time of complications in ICs |
| Profession | Perceived need for discussions at diagosis in patients | Chi-square (Fisher’s exact test) | p=0.050*, V=0.23 | 125 | Physicians were more likely than nurses and psycho-oncologists to report a need for discussions at diagnosis in patients |
| Profession | Perceived need for discussions at allo-HSCT indication in patients | Chi-square (Fisher’s exact test) | p=0.012*, V=0.27 | 125 | Psycho-oncologists were more likely than nurses and physicians to report a need for discussions at indication for allo-HSCT in patients |
| Profession | Perceived need for discussions on transplantation ward in patients | Chi-square (Fisher’s exact test) | p=0.025*, V=0.24 | 125 | Psycho-oncologists were more likely than nurses and physicians to report a need for discussions on transplantation ward in patients |
| Profession | Perceived need for discussions in outpatient setting in patients | Chi-square (Fisher’s exact test) | p=0.010*, V=0.31 | 125 | Psycho-oncologists were more likely than nurses and physicians to report a need for discussions in outpatient setting in patients |
| Profession | Perceived need for discussions at diagosis in ICs | Chi-square (Fisher’s exact test) | p=0.031*, V=0.23 | 125 | Physicians were more likely than nurses and psycho-oncologists to report a need for discussions at diagnosis in ICs |
| Profession | Perceived need for discussions at allo-HSCT indication in ICs | Chi-square (Fisher’s exact test) | p=0.048*, V=0.22 | 125 | Physicians were more likely than nurses and psycho-oncologists to report a need for discussions at indication for allo-HSCT in ICs |
| Profession | Perceived need for discussions at time of complications in ICs | Chi-square (Fisher’s exact test) | p=0.007*, V=0.29 | 125 | Physicians were more likely than nurses and psycho-oncologists to report a need for discussions at time of complications in ICs |
| Profession | Perceived need for discussions at change in therapy goal in ICs | Chi-square (Fisher’s exact test) | p=0.003*, V=0.30 | 125 | Physicians were more likely than nurses and psycho-oncologists to report a need for discussions at change in therapy goal in ICs |
| Profession | Perceived need for discussions at on transplantation ward in ICs | Chi-square (Fisher’s exact test) | p=0.005*, V=0.28 | 125 | Nurses were more likely than physicians and psycho-oncologists to report a need for discussions on transplantation ward in ICs |
| LAP-R | Perceived need for discussions at allo-HSCT indication in patients | Mann-Whitney-U | p=0.023*, u=0.24 | 103 | HCPs with higher death acceptance were less likely to report a need for discussion at indication for allo-HSCT in patients |
| DAP-R Escape Acceptance | Perceived need for discussions at allo-HSCT indication in ICs | Mann-Whitney-U | p=0.018*, u=0.26 | 81 | HCPs with higher escape acceptance were less likely to report a need for discussion at indication for allo-HSCT in ICs |
| DAP-R Death Avoidance | Perceived need for discussions at times of complications in patients | Mann-Whitney-U | p=0.034*, u=0.24 | 81 | HCPs with higher death avoidance were less likely to report a need for discussion at times of complications in patients |
| V = Cramér's V; u=effect size calculated as Z/√N; ρ=Spearman's rank correlation coefficient; Cohen’s g, *p≤0.05 | | | | | |

**Online Resource 9c) Associations and group differences in preferred professional group by sociodemographic and profession related variables in HCPs (n=125)**

| Variable | Outcome | Test | Result | n | Interpretation |
| --- | --- | --- | --- | --- | --- |
| Gender | Nurse preference | Chi-square | p = 0.049*, φ = 0.176 | 125 | Women being more likely to choose nurses as discussion partners |
| Gender | PC specialist preference | Chi-square | p=0.040*, φ =-0.184 | 125 | Women being more likely to choose PC specialists as discussion partners |
| Profession | Nurse preference | Chi-square (Fisher’s exact test) | p=0.002*, V=0.29 | 125 | Nurses being more likely to choose nurses as discussion partners |
| Age | PC specialist preference | Point-biserial correlation | r=-0.202, p=0.024 | 125 | Older HCPs were less likely to choose PC specialists as discussion partners |
| r = effect size (point-biserial correlation ), φ = Phi, V = Cramér's V, *p≤0.05 | | | | | |

**Online Resource 10: Comparison between chosen time points for discussions about life threat between patients and ICs**

|  | Patients (n=61) | | ICs (n=31) | |
| --- | --- | --- | --- | --- |
|  | Frequencies (n) | Percentages % | Frequencies (n) | Percentages % |
| Time of Diagnosis | 37 | 60.7% | 14 | 45.2% |
| Start of the first cancer therapy | 9 | 14.8% | 5 | 16.1% |
| Time of Indication for allo-HSCT | 3 | 4.9% | 2 | 6.5% |
| Shortly before admission to the transplantation ward | 1 | 1.6% | 1 | 3.2% |
| On the transplantation ward | 1 | 1.6% | 0 | 0.0% |
| Outpatient follow-up | 2 | 3.3% | 2 | 6.5% |
| In a situation without hope for a cure | 7 | 11.5% | 7 | 22.6% |
| No response | 1 | 1.6% | 0 | 0.0% |
| others | 0 | 0.0% | 0 | 0.0% |

**Online Resource 11: Factors influencing HCPs’ ideal and actual timing of conversation about life threat: results from logistic regression analyses**

| **Time Point** | **Predictor** | **B (SE)** | **Odds Ratio (Exp(B))** | **p-value** | **n** | **Interpretation** |
| --- | --- | --- | --- | --- | --- | --- |
| Time of Diagnosis (ideal) | Age  Gender  Escape Acceptance (DAP-R) | -0.053 (0.026)  1.078 (0.540)  -0.509 (0.236) | 0.949  2.938  0.601 | 0.043  0.046  0.031 | 123  123  81 | Older HCPs were less likely to consider diagnosis as ideal  Male HCPs preferred diagnosis  HCPs with a higher escape acceptance of death were less likely to choose this time point as ideal |
| Time of Indication for allo-HSCT (ideal) | Profession (physicians) | 1.545 (0.467) | 4.687 | <0.001 | 123 | Physicians preferred Indication significantly more than nurses |
| During the time of severe complications (ideal) | Age (older age) | -0.050 (0.024) | 0.952 | 0.041 | 123 | Older HCPs were less likely to consider this the ideal time for a conversation |
| During a change in goals of care (ideal) | Gender  Health Care Provision Center | 1.668 (0.672)  1.208 (0.602) | 5.304  3.348 | 0.013  0.045 | 123  123 | Men were more likely to consider this time point as ideal for conversation  HCPs working on the oncology ward were more likely to consider this time point as ideal compared to HCPs working on the HSCT ward |
| Patient initiates conversation (ideal) | Number of patients treated in the EOL-phase  Death Avoidance (DAP-R) | -0.034 (0.016)  -0.852 (0.269) | 0.967  0.427 | 0.035  0.002 | 123  81 | HCPs with more experience treating patients in the EOL phase were less likely to choose this time point as ideal  HCPs with a higher death avoidance were less likely to choose this time point as ideal |
| Time of Diagnosis (actually) | Profession  Profession  Approach Acceptance (DAP-R) | 4.249 (0.858)  3.263 (1.084)  -0.338 (0.176) | 70.025  26.118  0.713 | <0.001  0.003  0.055* | 123  123  81 | Physician are significantly more likely to actually discuss life threat at this time point compared to nurses  Psycho-oncologists are significantly more likely to actually discuss life threat at this time point compared to nurses  HCPs with a higher approach acceptance of death were less likely to actually discuss life threat at this time point (trend) |
| Time of Indication for allo-HSCT | Profession  Profession  Number of allo-HSCT patients treated | 2.688 (0.561)  2.401 (0.858)  0.009 (0.004) | 14.700    11.038  1.009 | <0.001  0.005  0.017 | 123  123  123 | Physician are significantly more likely to actually discuss life threat at this time point compared to nurses  Psycho-oncologists are significantly more likely to actually discuss life threat at this time point compared to nurses  HCPs with more experience treating allo-HSCT patients were less more to actually discuss life threat at this time point |
| Shortly before admission to the transplantation ward | Death Acceptance (LAP-R) | -0.151 (0.063) | 0.860 | 0.016 | 81 | HCPs with a higher death acceptance were less likely to actually discuss life threat at this time point |
| During the time of severe complications | Profession  Health Care Provision Center  Fear of Death (DAP-R) | 1.303 (0.471)  -0.995 (0.440)  -0.451 (0.212) | 3.681  0.370  0.637 | 0.006  0.024  0.033 | 123  123  81 | Physician are significantly more likely to actually discuss life threat at this time point compared to nurses  HCPs working on the oncology ward were more likely to consider this time point as ideal compared to HCPs working on the HSCT ward  HCPs with a higher fear of death were less likely to actually discuss life threat at this time point |
| During a change in goals of care | Profession  Fear of Death (DAP-R) | 1.298 (0.472)  -0.552 (0.243) | 3.629  0.576 | 0.006  0.023 | 123  81 | Physician are significantly more likely to actually discuss life threat at this time point compared to nurses  HCPs with a higher fear of death were less likely to actually discuss life threat at this time point |
| Patient initiates conversation | Death Avoidance (DAP-R) | -0.458 (0.218) | 0.663 | 0.036 | 81 | HCPs with a higher death avoidance were less likely to actually discuss life threat at this time point |

* p = 0.055, trend toward significance but did not reach the threshold of p < 0.05

**Online Resource 12:** **Participating centers and recruitment overview**

*Recruitment of HCPs*

HCPs were recruited at five German tertiary hospitals as part of CCCs:

- University Hospital Aachen
- University Hospital Bonn
- University Hospital Cologne
- University Hospital Düsseldorf
- University Hospital Leipzig

All five centers recruited HCPs.

*Recruitment of Patients and ICs*

Ten hematological centers across Germany were invited to participate in patient and IC recruitment:+

- University Hospital Cologne
- University Hospital Leipzig
- University Hospital Halle (Saale)
- University Hospital Schleswig-Holstein
- Technical University of Munich
- University Hospital Bochum
- University Hospital Berlin
- University Hospital Freiburg
- University Hospital Bonn
- University Hospital Frankfurt

Of these, seven centers ultimately recruited patient and/or IC participants. The following centers did not recruit patient or IC participants:

- University Hospital Freiburg
- University Hospital Bonn
- University Hospital Frankfurt
